# Early Enteral Nutrition Preserves Intestinal Barrier Function through Reducing the Formation of Neutrophil Extracellular Traps (NETs) in Critically Ill Surgical Patients

**DOI:** 10.1155/2020/8815655

**Published:** 2020-11-21

**Authors:** Qiongyuan Hu, Huajian Ren, Zhiwu Hong, Chenyang Wang, Tao Zheng, Yanhan Ren, Kai Chen, Song Liu, Gefei Wang, Guosheng Gu, Xiuwen Wu, Jianan Ren

**Affiliations:** ^1^Research Institute of General Surgery, Jinling Hospital, Medical School of Nanjing University, Nanjing 210002, China; ^2^Department of Gastrointestinal Surgery, Nanjing Drum Tower Hospital, The Affiliated Hospital of Nanjing University Medical School, 210008, China; ^3^Rosalind Franklin University of Medicine and Sciences, North Chicago, IL, USA

## Abstract

**Background:**

The gut was suggested as the driver of critical illness and organ injury. Recently, excessive formation of neutrophil extracellular traps (NETs) was associated with mucosal inflammation. Direct investigation of intestinal mucosa is essential to illuminate the potential mechanism of gut barrier in critically ill patients. We hypothesized that early enteral nutrition (EN) could decrease intestinal NETs and maintain the gut barrier.

**Methods:**

Intestinal biopsies were obtained using biopsy forceps from critically ill surgical patients complicated with enterocutaneous fistula. Expressions of tight junction (TJ) proteins, mucosal inflammation, and apoptosis were evaluated. Moreover, NET-associated proteins were evaluated in intestinal specimens of patients by Western blot and immunofluorescence analysis.

**Results:**

The intestinal barrier was significantly impaired in critically ill patients receiving early total parenteral nutrition (TPN), evidenced by intestinal villi atrophy, inflammatory infiltration, increased enterocyte apoptosis, and abnormal TJ expressions. Early EN significantly alleviated these intestinal injuries. In addition, we observed increased formation of the NET structure and elevated expressions of NET-associated proteins in intestines of critically ill surgical patients. Early EN was associated with the diminished presence of NETs and reduced expression of NET-associated proteins. Mechanically, analysis of the TLR4 pathway showed a significant increase in TLR4, NF*κ*B, and MAPK signaling in patients receiving TPN when compared to those receiving early EN.

**Conclusion:**

The intestinal barrier is disrupted in the human gut during critical illness. Our data suggests that an increased NET structure was showed in the gut of critically ill surgical patients, and early EN treatment was associated with the reduction of NET formation and the preservation of mucosal immunity.

## 1. Background

Critical illness disrupts gut integrity by increasing inflammatory response and epithelial apoptosis. The gut has long been suggested as the “engine” of multiple organ dysfunction (MOD) for critically ill patients [1]. However, the original evidence for this theory has only been supported by experimental or retrospective data. Direct investigation of human intestinal mucosa in critically ill patients is required to confirm this theory. Increasing evidence also suggested that an advanced approach to target the human gut would be the most effective way of demonstrating the gut's role as the driver of critical illness [1, 2].

Nutritional support is the cornerstone of therapy for critical illness. In the absence of enteral nutrition (EN), disruption of mucosal integrity contributes to an impaired gut barrier [3, 4]. Increasing evidence based on animal models demonstrated that total parenteral nutrition (TPN) contributes to increased infection incidence due to mucosal injury [5]. However, changes in the gut barrier and mucosal morphology of critically ill patients receiving TPN or EN are scarcely documented and poorly understood. Piton et al. [6] recently investigated the impact of early EN on intestinal mucosa in shock patients by measuring plasma biomarkers of enterocyte damage. However, the precise mechanism of the impact of nutrition on the intestinal barrier of critically ill patients still remains undetermined. Therefore, direct evaluation of the intestine in critically ill patients is essential to deeply understand the potential mechanisms of gut dysfunction.

Accumulated neutrophils at the subepithelial space can disrupt the local immunological milieu and impair the epithelial barrier. In 2004, a new mechanical action was described in neutrophils: activated neutrophil can erupt web-like chromatin filaments to form neutrophil extracellular traps (NETs) in response to various states of inflammation [7]. A recent study demonstrated that NETs are implicated in the inflammatory cascade of ulcerative colitis patients [8]. Increased inflammatory response in the gut is very crucial in the progression of patients with sepsis and MOD [9]. The contribution of NETs to intestinal inflammation and gut barrier dysfunction remains to be elucidated. Therefore, we compared the effects of the formation of NET structures in critically ill patients receiving either EN and TPN.

The primary objective of our study was to investigate changes in intestinal mucosa during critical illness among patients receiving EN or TPN. The secondary objective is aimed at evaluating the formation of the NET structure in the gut among critically ill patients and comparing the impact of different routes of nutrition on NET formation.

## 2. Materials and Methods

### 2.1. Ethics Statement and Human Tissues

Our study was performed based on the Recommendations of Guidelines for Clinical Trials by the Ethics Committee of Jinling Hospital. All patients provided written informed consent before performance of any study-related procedure.

**Figure 1:**
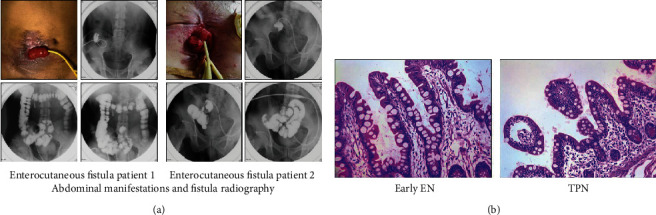
The effect of early EN on mucosal morphology in critical illness. (a) Abdominal manifestations and fistula radiography for critically ill surgical patients. (b) H&E staining for intestinal histology in critical illness receiving different types of nutrition.

Recently, the evaluation of gut barrier function by measuring circulating biomarkers for critically ill patients has been limited and indirect. In addition, there is no consensus on the appropriate cutoff points for plasma biomarkers [10]. In our study, we presented an ideal model (enterocutaneous fistula (ECF)) to directly observe the morphology and investigate the intestines during critical illness. Abdominal manifestations and fistula radiography for ECF patients are shown in Figure 1(a). Intestinal mucosa specimens were percutaneously obtained using biopsy through the fistula. Informed consent was obtained from all participants that received this procedure.

Our prospective study cohort enrolled 20 ECF patients who were admitted to the surgical intensive care unit (SICU) at Jinling Hospital from October 2018 to Jun 2019. The SICU, which belongs to the Department of General Surgery, receives abdominal trauma and perioperative patients. For critical illness, although early EN is preferred, the TPN group is still used in some specific circumstance, including (a) inability to tolerate adequate caloric intake, (b) continued vomiting, (c) increasing abdominal distension, (d) evidence of intestinal obstruction, (e) intra-abdominal infections complicated with other undefined fistula, and (f) high-output fistula. Feeding routes were determined by chart review and discussion with the family and the surgical team. In our study, the groups were divided according to the routes of nutrition: one group received early TPN (TPN group, *n* = 10), whereas the other group received early full enteral nutrition (early EN group, *n* = 10) for seven days. On day 7, intestinal samples were obtained. The baseline characteristics of the enrolled patients are shown in Supplementary Table 1. At baseline, there was no difference between the early EN and TPN groups.

### 2.2. Histology, Immunohistochemistry, and Immunofluorescence

Human intestine biopsies were fixed in formalin and embedded in paraffin. Hematoxylin and eosin (H&E) staining was then performed, and Chiu's scoring system was used to evaluate intestinal injuries. To evaluate the density of goblet cells, periodic acid-Schiff (PAS) staining and Alcian blue-PAS staining were performed, as previously described [11].

For immunohistochemistry (IHC) staining, slides were incubated with anti-IL-1*β* antibody (Servicebio, GB11113, Wuhan), anti-IL-6 antibody (Servicebio, GB11117-1, Wuhan), anti-TNF-*α* antibody (Servicebio, GB11188, Wuhan), anti-Bax antibody (Servicebio, GB11007-1, Wuhan), and anti-Bcl-2 antibody (Servicebio, GB12318, Wuhan) after antigen retrieval (with citrate buffer) and organization background closing (with goat serum). Then, the slides were incubated with a biotin-linked second antibody (goat-anti-rabbit) for 20 min, with streptavidin labeled with horseradish-peroxidase (S-A/HRP) for 20 min and with DAB (Servicebio, K5007, Wuhan) for 5 min [12]. The sections were stained with hematoxylin and then dehydrated. Finally, the staining was observed under a microscope.

The localization and expression of tight junction (TJ) proteins and NET-associated proteins were detected by immunofluorescent staining using antibodies against ZO-1 (ab96587; Abcam), occludin (ab216327, Abcam), claudin-1 (ab180158, Abcam), mouse anti-human MPO (ab25989, Abcam), rabbit anti-human NE (ab131260, Abcam), and rabbit anti-human citH3 (ab5103, Abcam). DAPI was used to counterstain DNA. Images were acquired using a Leica DMI 4000 B fluorescence microscope.

### 2.3. Western Blot

For this assay, 0.1 g of intestinal samples was homogenized and ultrasonicated and then treated with lysis buffer. All protein samples were boiled after being mixed with the commensurable buffer. The expression level of proteins from intestines was detected and separated via SDS-PAGE gels. The total protein from gels was then transferred to PVDF membranes. The membranes were blocked with milk and then incubated with primary antibodies against the protein of interest at 4°C overnight. Next, the membranes were incubated with HRP-labeled secondary antibodies. The expression of protein was quantified in optical density units using Image Lab software (Bio-Rad, CA, USA) and was normalized to the corresponding sample expression of GAPDH (P.R.C. KeyGEN Biotech).

### 2.4. Apoptosis Evaluation

The detection of apoptosis in the intestinal tissues used TUNEL (terminal deoxynucleotidyl transferase dUTP nick end labeling) staining according to the manufacturer's instruction, as previously described [13].

### 2.5. Statistical Analysis

Statistical analyses were performed using SPSS software. Results are expressed as means ± SD unless otherwise specified. A comparison between two groups used the unpaired, one-tailed Student *t*-test. For the expression of protein and cytokines, a paired Student *t*-test was used. All *p* values < 0.05 were considered significant.

## 3. Results

### 3.1. The Effect of Early EN on Mucosal Morphology and Biochemical Barrier in Critical Illness

Morphologically, villus atrophy and decline in villus height were shown in the TPN group, and early EN improved villus morphology (Figure 1(b)). Furthermore, H&E staining of intestines showed significant intestinal mucosal injury, evidenced by tissue destruction and infiltration of inflammatory cells in patients receiving TPN (Figure 1(b)). However, early EN significantly alleviated these injuries. Additionally, using PAS and Alcian blue-PAS staining, our results showed a significant increase in the number of goblet cells in the early EN group compared with that of the TPN group (Supplementary Figure 1).

### 3.2. Early Enteral Nutrition Improves the Immunological Milieu in the Gut

**Figure 2:**
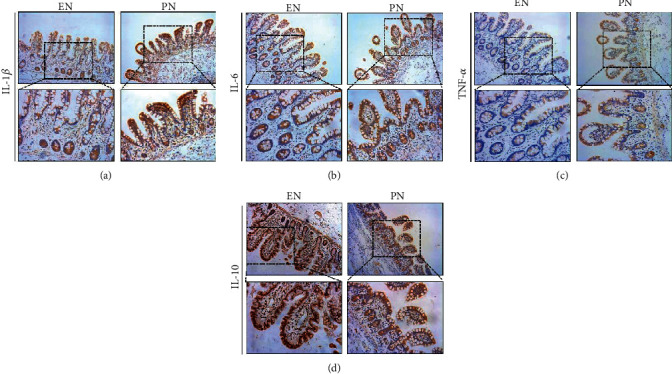
Early enteral nutrition improves the immunological milieu in the gut. Representative immunohistochemical images showing the inflammatory factors, IL-1*β*, IL-6, TNF-*α*, and IL-10, in intestinal samples taken during critical illness.

A dense network of immune cells that cooperate with the gastrointestinal epithelium maintains inflammatory balance in the gut [5]. IHC staining showed that proinflammatory factors, including IL-1*β*, IL-6, and TNF-*α*, were significantly increased in the TPN group compared with the early EN group (Figures 2(a)–2(c)). The anti-inflammatory cytokine IL-10, mainly derived from intraepithelial lymphocytes, is a vital factor for the intestinal epithelial barrier.  Figure 2(d) showed that IL-10 expression was markedly decreased in the TPN group compared with the EN group.

### 3.3. Early EN Restores Intestinal Physical Barrier for Critically Ill Patients

**Figure 3:**
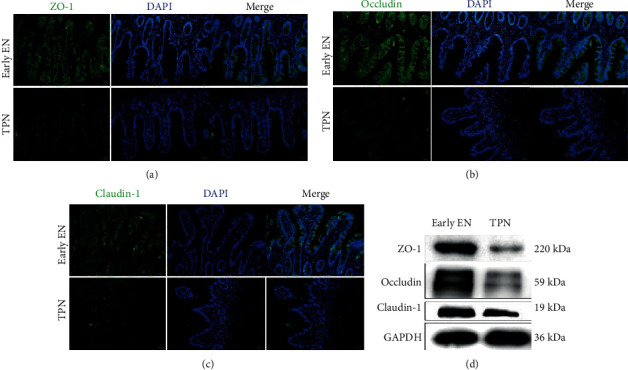
Effect of early enteral nutrition on the intestinal mechanical barrier. (a) Location and expression of tight junction protein within intestinal mucosa in critically ill patients, comparing TPN and early EN groups. (b) Protein levels of ZO-1, occludin, and claudin-1 were determined using Western blotting.

Impaired TJ integrity is the key factor for increased intestinal epithelial permeability [12]. Our results showed that the expressions of ZO-1, occludin, and claudin-1 were significantly increased in the intestinal mucosa in the early EN group compared with those of the TPN group (Figure 3). The presence of impaired intestinal villi was further verified through microscopy. As shown in Supplementary Figure 2, the ultrastructural morphology of TJ and desmosomes in the intestines was disrupted in the TPN group. However, the TJ and desmosomes were nearly intact in patients receiving EN.

**Figure 4:**
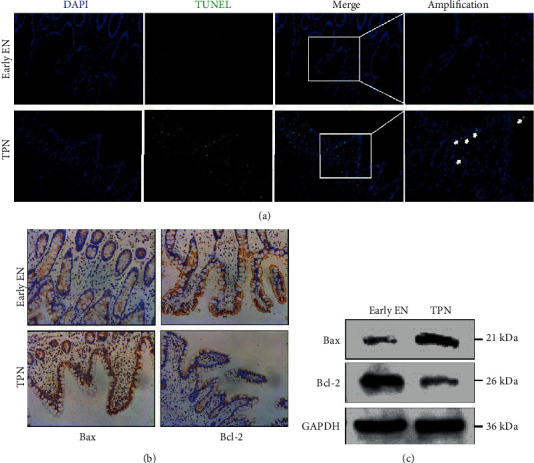
The effect of early nutrition on intestinal apoptosis. (a) Representative images of TUNEL staining in the gut of critically ill patients. (b) Representative immunohistochemical images showing the Bax and Bcl-2 expression in the intestinal samples of critically ill patients. (c) The expression of Bax and Bcl-2 was determined by Western blotting. GAPDH was used as control.

Enterocyte apoptosis is one of the important accelerants of disrupted TJ [13]. An increased number of TUNEL-positive cells were shown in the gut mucosa in the TPN group, while early EN treatment significantly attenuated apoptosis of enterocytes (Figure 4(a)). Moreover, the expression of the proapoptotic protein Bax and antiapoptotic protein Bcl-2 was assessed to check the apoptosis level in the gut. IHC staining and Western blot results showed increased expression of Bax and decreased expression of Bcl-2 in the TPN group as compared to the early EN group (Figures 4(b) and 4(c)).

### 3.4. NET-Associated Proteins Are Upregulated in the Gut of Critically Ill Patients and Could Be Diminished following Early EN Treatment

**Figure 5:**
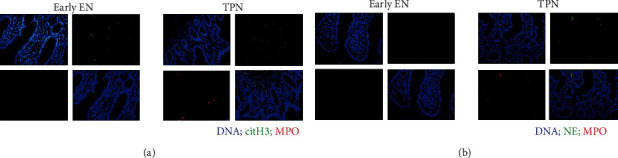
Early enteral nutrition reduces the intestinal presence of NETs. (a) Representative images of double immunofluorescence staining of intestinal samples in critically ill patients for citrullinated histone H3 (citH3, green) and myeloperoxidase (MPO, red). (b) Double immunofluorescence staining for neutrophil elastase (NE, green) and myeloperoxidase (MPO, red). Colocalizations of citH3 and MPO or NE and MPO are indicative of NET structure.

Increased formation of NETs is associated with gut damage. However, the clinical plasma biomarkers of NETs are unstable and subject to enzymatic degradation [14]. Better detection of local formation of the NET structure is required to investigate the potential mechanisms. Microscopic analysis detected positive staining for citH3 and MPO (Figure 5(a)) and NE and MPO (Figure 5(b)), overlapping with diffuse DNA scaffolds and confirming the increased formation of the NET structure in the gut during critical illness.  Figure 5 also showed that early EN is associated with the diminished presence of NETs in the intestinal mucosa. PAD4 acts as a driver of NET formation, and Western blot demonstrated a significant decrease in the gut in the early EN group as compared to the TPN group (Supplementary Figure 3). We next evaluated the other three components of NETs, including the expression of NE, MPO, and citH3. The expression of these NET-associated proteins was decreased in the intestines of patients receiving early EN.

### 3.5. Effects of Early EN on the TLR4 Pathway

The expression of TLR4 protein in the gut was assessed in our patients. The results showed that early EN was associated with decreased TLR4 expression in the intestines compared with that of the TPN group. The NF*κ*B pathway activated by TLR4 could initiate the transcription of inflammatory genes. Supplementary Figure 4A showed that p-IKK*β*, p-I*κ*B*α*, and p-p65 proteins were greatly reduced in the early EN group compared with those of the TPN group. MAPKs are another vital pathway activated by TLR4. The p38, ERK, and JNK signaling was evaluated using Western blot. Our results indicated that the levels of p-p38 and p-ERK were significantly decreased in the gut in the early EN group (Supplementary Figure 4B). Interestingly, the expression of p-JNK protein showed no significant differences between the two groups.

## 4. Discussion

The gut has long been suggested to be the driver of critical illness [1]. Under normal physiological conditions, the gut plays an important role in health maintenance. However, multiple elements of the gut are damaged in critically ill patients, potentially worsening MOD. Our study examined potential mechanisms for these changes and provided solid proof for the importance of the gut in human critical illness. We investigated the changes in the intestinal barrier in critically ill patients receiving different types of early nutrition. Our results demonstrated that the patients receiving TPN showed an injured mucus barrier, increased inflammatory response, abnormal TJ proteins, and increased apoptosis in the intestinal specimens compared with the enterally fed gut. This finding correlated with increased formation of the NET structure in the gut. Excessive formation of NETs was recently suggested to contribute to mucosal injury [15, 16]. Immunofluorescence images demonstrated that the intensity and distribution of the NET structure were significantly increased in the gut of critically ill patients receiving TPN. Therefore, the dysfunction of the gut barrier may be associated with increased NET-associated proteins and NET structure.

In the present study, atrophic mucosa was also shown in the patients with TPN treatment; early EN treatment significantly helped to restore the mucosal morphology. These findings indicate that an interaction between intestinal epithelial cells and nutrients plays an important role in keeping cellular function and metabolism active. In one study of rats with brain injury, unfed rats, compared to those with delayed EN, showed a significant atrophic mucosa and faster apoptosis of epithelial cells. Additionally, restarting EN was suggested to awake dormant intestinal mucosal cells, which can induce epithelial cells to enhance protein synthesis [17]. Mucin secreted by goblets also plays an important role to protect the gut epithelium against bacteria invasion. In the present study, early EN treatment helped to maintain the number of goblets in the human gut during critical illness.

Results from studies of mice and cell culture suggested that impaired TJ proteins were associated with the increased production of proinflammatory cytokines and were elevated following TPN treatment. Our previous study suggested that increased production of proinflammatory cytokines contributes to increased epithelial cell apoptosis [13]. In the present study, we confirmed that increased local proinflammatory factors in the human gut were associated with alternant expression of TJ proteins and increased apoptosis in patients receiving TPN. These significant changes in the production of proinflammatory factors initiate a chain effect leading to a profound impairment of the cellular mechanisms that preserve the intestinal epithelial barrier under physiological conditions [18]. Anti-inflammatory responses that are regulated by IL-10 are important in maintaining the epithelial barrier. Evidence obtained from in vivo studies of mice indicated that IL-10 expression is markedly decreased during TPN, which is associated with injured TJs and increased epithelial permeability. Sun et al. [19] showed that the exogenous administration of IL-10 markedly alleviated the TPN-associated decrease in TJ protein expression. In our study, EN deprivation was associated with decreased levels of IL-10, and early EN treatment significantly increased IL-10 expression in the gut of critically ill patients.

Recent studies investigated that excessive formation of NET structures was suggested to lead to the progression of sepsis or MODS [20]. Elevated levels of NET biomarkers have been reported in critically ill patients, but currently, methods to detect NET formation are limited to indirect measurement in the circulation [21, 22]. Moreover, circulating levels of NET markers are unstable and subject to enzymatic degradation. Therefore, detection of local NET formation is urgently necessary for the study of critical illness. Recently, NETs were suggested to directly induce epithelial cell death [23], which can aggravate gut barrier dysfunction. Lang et al. [24] reported that the degradation of NETs could reverse intestinal inflammation and injury in nonobese diabetic mice. Additionally, Dinallo et al. [8] showed that NETs were released in colonic mucosal tissue and suggested that excessive induction of NETs contributes to the amplification of intestinal inflammatory cascade. Li et al. [16] recently demonstrated that NET degradation by DNase may be protective in DSS-induced experimental colitis, and DNase treatment prevented the increase in serum inflammatory cytokines. In the present study, we discovered that the gut of critically ill patients showed increased protein levels of NE and MPO, two main components of NETs. Our IF images further showed that NE and MPO colocalize with DNA and citH3, indicating that the NET structure occurs in the gut of critically ill patients. Accordingly, a diminished presence of the NET structure was shown in early EN-treated patients. Further investigation is required to delineate the potential mechanisms involved in the clearance of intestinal NETs.

TLR4 signaling is an important pathway by which the intestinal mucosa senses change in the luminal environment. Our study found that the expression of TLR4 was significantly increased in the gut of critically ill patients receiving early TPN as compared with early EN. There is an emerging theory that altered microbial populations in patients receiving TPN induce intestinal barrier dysfunction, including an increase in many Gram-negative organisms. Nutrients play an important role in regulating intestinal bacteria-mucosal immune crosstalk [25, 26]. An expansion of Gram-negative bacteria has been suggested to activate the TLR4 signaling pathway [27]. Although we did not report microbial data, the findings of increased TLR4 expression would be consistent with previous publications. Recent studies indicated that the TLR4 signaling pathway is associated with the increased formation of NET structures [28]. In this study, TLR4 signaling was likely involved in intestinal NET formation.

The main limitation of this study is that all patients suffered ECFs, and this affects enterohepatic circulation that also affects gut architecture. Additionally, all the patients were critically ill, and because of associated conditions/morbidity, there may have been an additional effect on the enterocytes. However, it is really difficult and dangerous to perform systematic digestive endoscopy in critically ill patients. We provide an advanced approach to evaluate the pathological changes in the gut using the ECF model.

The concept of the gut as the driver of critical illness has been established for more than 30 years. The dysregulation of the intestinal microenvironment can drive the progression of sepsis and induce distant organ injury. However, no current therapy exists to target the gut barrier or permeability at the bedside of critically ill patients. The clinical model (ECF) in our study helps to investigate the impact of selected pharmacologic therapies on the local intestinal mucosa, while further studies are urgently required to determine how to target mucosa during critical illness.

## Data Availability

The data used to support the findings of this study are included within the article.

## Supplementary Materials

Supplementary Materials Supplementary Table 1: demographic and clinical variables of patients in surgical ICU. Supplementary Figure 1: effect of early nutrition on the biochemical barrier in the gut. (A) PAS staining and AB-PAS staining were performed to determine the numbers of intestinal goblets in the gut. Supplementary Figure 2: critical illness damaged the morphology of tight junctions in the gut. (A) In the intestinal samples in patients receiving TPN, the TJ ultrastructure was injured. The TJs were nearly intact in the gut of the early EN group. Supplementary Figure 3: expression of NET-associated proteins was determined in the gut of critically ill patients. Western blot showing PAD4, NE, MPO, and citH3 expression in the intestinal samples, comparing the TPN and early EN groups. Supplementary Figure 4: effect of early enteral nutrition on the expression level of TLR4 signaling. (A) The levels of TLR4, IKK*β*, I*κ*B*α*, and p65 protein were analyzed in the gut in critically ill patients receiving different types of nutrition. (B) Effect of nutrition on MAPK signaling, including p38, ERK, and JNK, in the human intestine during critical illness.
