# Supplementary material for: Early Enteral Nutrition Preserves Intestinal Barrier Function through Reducing the Formation of Neutrophil Extracellular Traps (NETs) in Critically Ill Surgical Patients
Source: Oxid Med Cell Longev. 2020 Nov 21;2020:8815655. doi: 10.1155/2020/8815655 (PMC7700037; doi:10.1155/2020/8815655)

Supplementary Table 1 Demographic and clinical variables of patients in surgical ICU.

| Parameters | Critically ill surgical patients | | p value |
| --- | --- | --- | --- |
|  | Early EN (n=10) | TPN (n=10) |  |
| Age, mean (SD), y | 51.3 ± 15.7 | 53.3 ± 16.8 | 0.786 |
| Gender (male), n (%) | 8 (80.0%) | 7 (70%) | 0.267 |
| BMI, mean (SD), (kg/m^2^) | 21.5 ± 3.1 | 21.3 ± 2.8 | 0.881 |
| Primary disease, n (%) |  |  | 0.410 |
| Traffic accident | 1 (10%) | 2 (20%) |  |
| Injury **^a^** | 2 (20%) | 2 (20%) |  |
| Surgical complication **^b^** | 7 (70%) | 6 (60%) |  |
| Site of infection, n (%) |  |  | 0.267 |
| Abdominal | 7 (70%) | 8 (80%) |  |
| Pulmonary | 3 (30%) | 2 (20%) |  |
| **ICU admission** |  |  |  |
| APACHE II score, mean (SD) | 9.8±4.5 | 11.2±4.9 | 0.514 |
| SOFA sore, mean (SD) | 5.4±2.9 | 6.5±3.4 | 0.446 |
| CRP, mean (SD), mg/L | 59.6 ± 29.2 | 65.7 ± 33.2 | 0.668 |
| PCT, mean (SD), ng/mL | 3.1 ± 1.5 | 3.6 ± 2.0 | 0.535 |

BMI: body mass index; APACHE: Acute Physiology and Chronic Health Evaluation; SOFA: Sepsis-related Organ Failure Assessment; CRP: C-reaction protein; PCT: procalcitonin. **^a^**Injury includes gunshot, falling, cuts, and bruising; **^b^**Patients who developed into intra-abdominal infection after elective surgery were categorized as having surgical complication.

**Supplementary Figure 1** Effect of early nutrition on biochemical barrier in the gut. (A) PAS staining and AB-PAS staining were performed to determine the numbers of intestinal goblets in the gut.


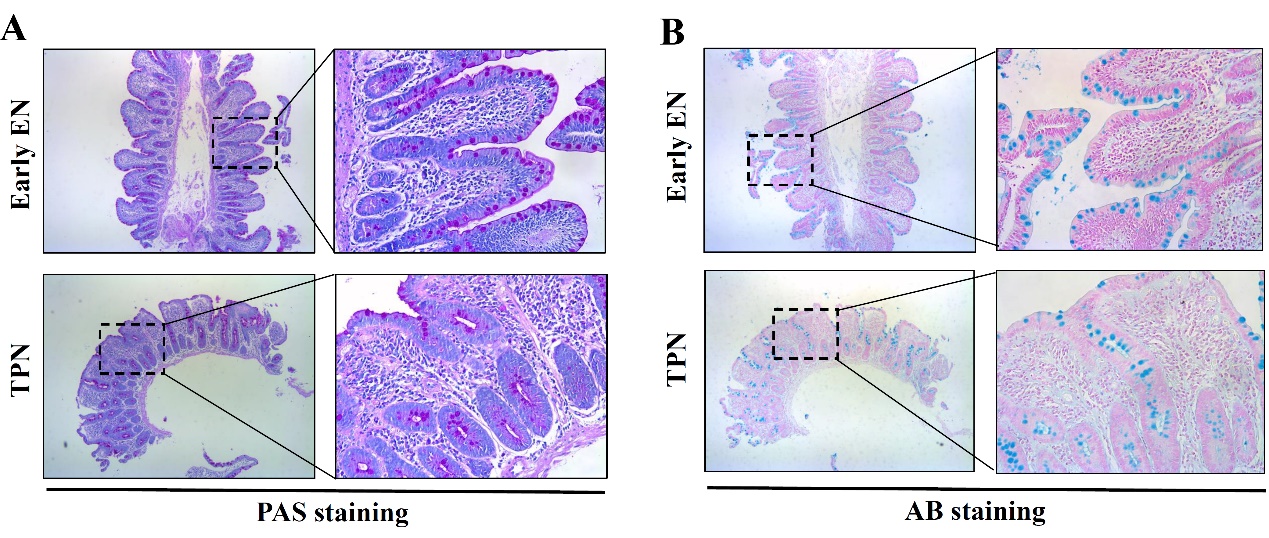


**Supplementary Figure 2** Critical illness damaged the morphology of tight junctions in the gut. (A) In the intestinal samples in patients receiving TPN, the TJs ultrastructure were injured. The TJs were nearly intact in the gut of the early EN group.


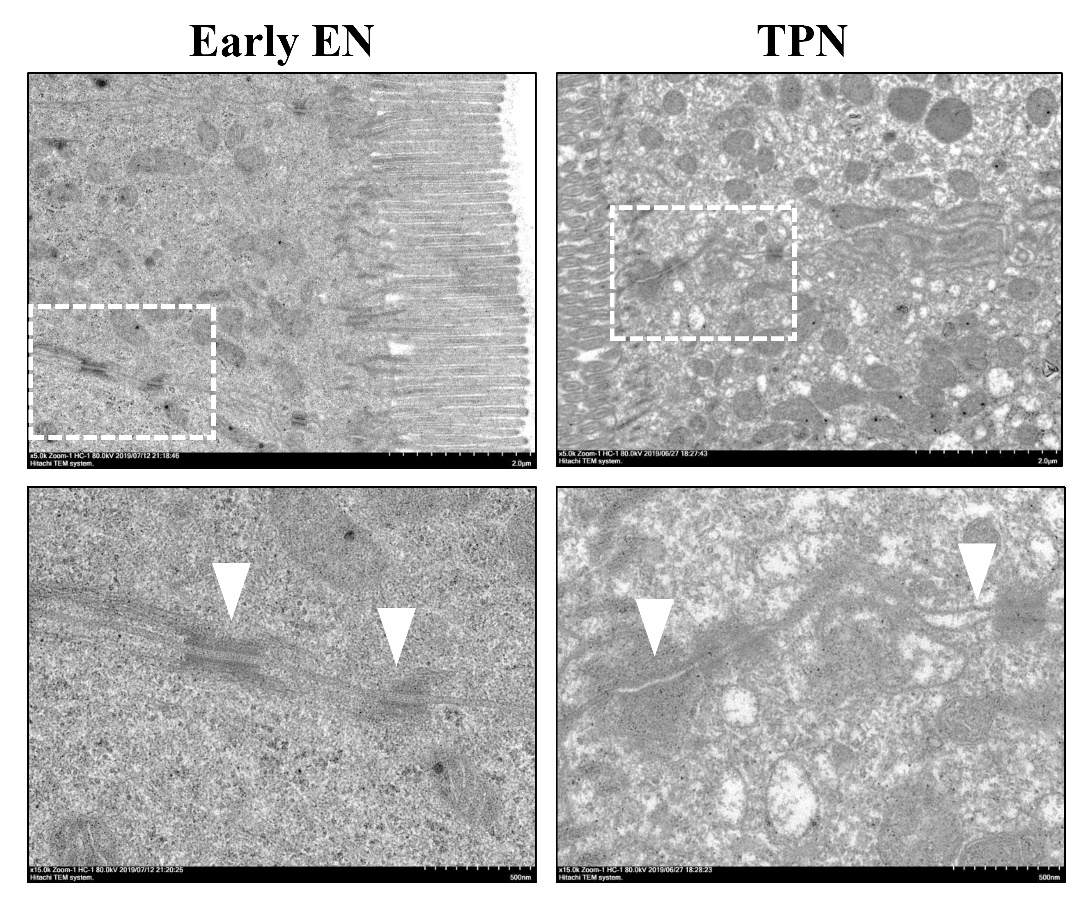


**Supplementary Figure 3** Expression of NETs-associated proteins were determined in the gut of critically ill patients. Western blot showing PAD4, NE, MPO, and citH3 expression in the intestinal samples, comparing TPN and early EN group.


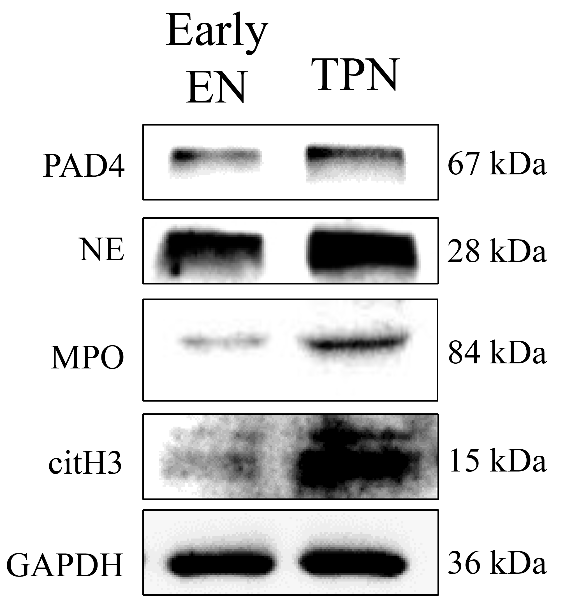


**Supplementary Figure 4** Effect of early enteral nutrition on the expression level of TLR4 signaling. (A) The level of TLR4, IKKβ, IκBα, and p65 protein were performed in the gut in critically ill patients receiving different types of nutrition. (B) Effect of nutrition on MAPKs signaling, including p38, ERK, and JNK, in human intestine during critical illness.


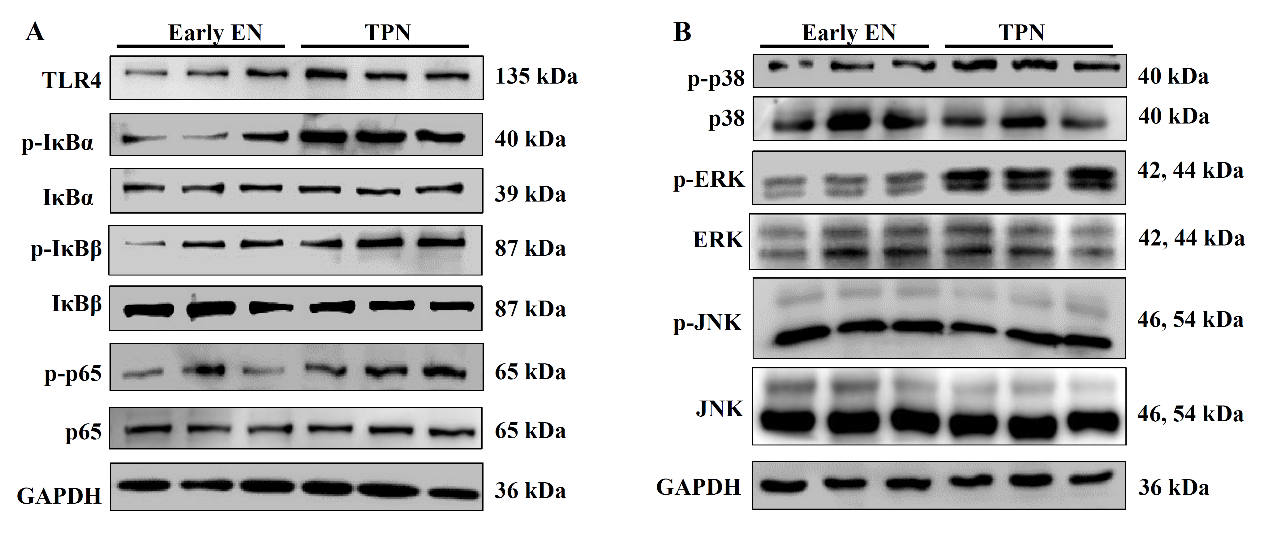

Supplement: Supplementary Materials — Supplementary Table 1: demographic and clinical variables of patients in surgical ICU. Supplementary Figure 1: effect of early nutrition on the biochemical barrier in the gut. (A) PAS staining and AB-PAS staining were performed to determine the numbers of intestinal goblets in the gut. Supplementary Figure 2: critical illness damaged the morphology of tight junctions in the gut. (A) In the intestinal samples in patients receiving TPN, the TJ ultrastructure was injured. The TJs were nearly intact in the gut of the early EN group. Supplementary Figure 3: expression of NET-associated proteins was determined in the gut of critically ill patients. Western blot showing PAD4, NE, MPO, and citH3 expression in the intestinal samples, comparing the TPN and early EN groups. Supplementary Figure 4: effect of early enteral nutrition on the expression level of TLR4 signaling. (A) The levels of TLR4, IKKβ, IκBα, and p65 protein were analyzed in the gut in critically ill patients receiving different types of nutrition. (B) Effect of nutrition on MAPK signaling, including p38, ERK, and JNK, in the human intestine during critical illness. [file 8815655.f1.docx]
